# Halovirus HF2 Intergenic Repeat Sequences Carry Promoters

**DOI:** 10.3390/v13122388

**Published:** 2021-11-29

**Authors:** Brendan Russ, Friedhelm Pfeiffer, Mike Dyall-Smith

**Affiliations:** 1 Department of Microbiology, Clayton Campus, Monash University, Clayton 3800, Australia; brendan.russ@monash.edu; 2 Computational Biology Group, Max-Planck-Institute of Biochemistry, 82152 Martinsried, Germany; fpf@biochem.mpg.de; 3 Veterinary Biosciences, Faculty of Veterinary and Agricultural Sciences, University of Melbourne, Parkville 3010, Australia

**Keywords:** halovirus, haloarchaea, halobacteria, *Halorubrum coriense*, transcription, promoter

## Abstract

Halovirus HF2 was the first member of the *Haloferacalesvirus* genus to have its genome fully sequenced, which revealed two classes of intergenic repeat (IR) sequences: class I repeats of 58 bp in length, and class II repeats of 29 bp in length. Both classes of repeat contain AT-rich motifs that were conjectured to represent promoters. In the present study, nine IRs were cloned upstream of the *bgaH* reporter gene, and all displayed promoter activity, providing experimental evidence for the previous conjecture. Comparative genomics showed that IR sequences and their relative genomic positions were strongly conserved among other members of the same virus genus. The transcription of HF2 was also examined by the reverse-transcriptase-PCR (RT-PCR) method, which demonstrated very long transcripts were produced that together covered most of the genome, and from both strands. The presence of long counter transcripts suggests a regulatory role or possibly unrecognized coding potential.

## 1. Introduction

HF2 is a myovirus belonging to the species *Haloferacalesvirus HF1,* within the genus *Haloferacalesvirus* (family: *Hafunaviridae*, previously *Myoviridae*) [1]. It is lytic and can infect two species of extremely halophilic archaea, *Halorubrum saccharovorum* and *Halorubrum coriense* [2]. The viral genome is linear dsDNA, 77,672 bp in length and has a GC content of 56%, significantly lower than that of its host species (67%). It was the first halovirus genome to be fully sequenced [3] revealing a gene organization typical of myoviruses, but with many of its 127 annotated genes coding for proteins of unknown function. It also carries four tRNA genes. The genomic termini consist of 306 bp direct repeats, and replication proceeds by concatemer formation, followed by precise cutting at a specific target sequence [4]. The presence of direct terminal repeats (DTR) is reminiscent of the genome structure of coliphage T7 and related viruses [5].

The modular gene organization in haloferacalesviruses is well conserved [1,6]. Genes are organized as modular units in two genomic arms so that most genes are directed towards the centre. Many of the genes of the left arm have unknown function but some are involved in replication, DNA modification and accessory biological activities, while the right arm carries genes for virus morphogenesis and DNA packaging. Recombination events can replace large parts of the genome, as was revealed from a comparison of HF2 and the related virus, HF1 [7]. The two viruses differ in genes coding for proteins of virion morphogenesis, and the divergent tail genes are consistent with their differing host ranges: HF1 has a broad host range, being able to infect haloarchaea of at least three genera (*Haloferax*, *Halobacterium* and *Halorubrum*), while HF2 has a much narrower host range, and infects two species of *Halorubrum* [2,7].

The HF2 transcriptome was first studied by Tang et al. [3], who used Northern blot hybridisation to identify and map transcripts at various times post-infection (p.i.). Consistent with the orientation of gene modules, the majority of the HF2 genome is transcribed inwards from the termini, with transcript lengths ranging from 0.5 to 9 kb, and often overlapping. The replication cycle of HF2 in *Hrr. coriense* is approximately 5 h, with groups of transcripts being synthesised from particular regions of the genome in a temporal pattern of early (0–1 h p.i.), middle (1–3 h p.i.) and late (3–5 h p.i.). Early and middle transcripts were produced from the left arm of the genome, while late transcripts were produced from genes of the virus morphogenesis module found at the right arm of the genome. This pattern of gene expression is similar to that found in many well studied bacterial caudoviruses, such as T7 [5].

Intriguingly, the 5′ ends of several transcripts occurred at the approximate location of intergenic repeat sequences (IRs) [3,6]. IRs fall into two distinct sequence groups (class I and II), and the members of each class are found in different regions of the genome. Class I IRs are 56–58 bp long and occur in the first 49 kb of the genome among genes that are transcribed from the early to middle stages of virus infection. While most of the genes in this region cannot be assigned a function, it includes genes involved in DNA synthesis and replication (e.g., helicase, DNA polymerase, ribonucleotide reductase), tRNA repair and DNA methylation. Class II repeats are 29 bp long and restricted to the right arm of the genome (54–77 kb), within the virus morphogenesis module that is transcribed late in infection.

Although many new isolates and genome sequences of haloviruses have recently become available [1], little is known regarding the regulation of gene expression during lytic infection or in the provirus state. Within both classes of IRs are AT-rich sequences that could represent promoter motifs, but experimental evidence supporting this hypothesis has so far been lacking. The aims of this study were to test whether the IRs of HF2 contain functional promoters; to map the 5′ ends of IR-directed transcripts; and to search for long transcripts that would have escaped detection by the previous method of Northern blot analysis.

## 2. Materials and Methods

### 2.1. Strains and Cultivation

Conditions for growth of the Δ*radA* strain of *Hfx*. *volcanii*, DS52, were reported previously [8]. *Escherichia coli* strain DH5α (*recA1*, *endA1*, *mcrA*+, *mcrB*+, *gyrA96*) from Stratagene (La Jolla, CA, USA), was used for plasmid propagation and transformation, and was cultured in Luria-Bertani medium supplemented with ampicillin (100 μg/mL) where necessary. Transformation of *E*. *coli* was by the TSS method [9]. while *Hfx*. *volcanii* was transformed as previously described [10]. Cultures of *Hrr. coriense* were maintained in 18% Modified Growth Medium (MGM; 2.47 M NaCl, 90 mM MgCl_2_, 90 mM MgSO_4_, 60 mM KCl, 3 mM CaCl_2_, 10 mM Tris-HCl pH 7.5, 5 g/L bacteriological peptone, 1 g/L yeast extract. The pH was adjusted to 7.5 with Tris base). Cultures were incubated at 37 °C, with orbital shaking (180 rpm), and supplemented with novobiocin (0.3 μg/mL), when required to maintain plasmids.

### 2.2. Reporter Plasmids pRV1 and pRV2

The reporter plasmid pRV1 was used in the study by Large et al. [11] to measure promoter strength in *Hfx. volcanii*, but its construction has not been previously described. It was developed from the 13.3 kb plasmid pMLH32 [12] which carries *E.coli* genes for replication and ampicillin resistance (*bla*) from plasmid pBS(+); the replication region of haloarchaeal plasmid pHK2 from *Hfx. lucentense*; the novobiocin resistance gene *gyrB* (*Hfx. lucentense*); and the gene for β-galactosidase BgaH (*bgaH*; *Hfx. lucentense*). The existing *Nde*I site and one *Eco*RI site of pMLH32 were removed by partially cutting with *Kpn*I (there are two sites) followed by complete digestion with *Nde*I. The exposed termini were blunted with T4 DNA polymerase, the ends joined by ligation, the products transformed into *E.coli* and transformants grown under ampicillin selection. From these, a colony carrying the desired plasmid was selected, in which the *Kpn*I and *Nde*I sites at nt 1 and 712 were destroyed and the intervening fragment removed, which contained an *Eco*RI site (adjacent to the *Kpn*I site) as well as the f1 *ori* and *lacZα* genes from the *E.coli* plasmid pBS+. 

A second *Eco*RI site, within the *gyrB* gene, was removed by site-directed mutagenesis, to alter GAATTC (nt 7823–7828) to GAATT**G**, producing a silent change (GGG Gly to GGC Gly) within the *gyrB* ORF. Next, the *Hind*III-*Eco*RI fragment containing the 5′ end of *bgaH* was replaced with a PCR modified version of the same sequence that contained an *Nde*I site positioned at the start codon of the *bgaH* ORF (i.e., nt 2614–2629: TTGTGT ATG ACA GTT changed to TTG**CA**T ATG ACA GTT). Then, a 97 nt sequence (nt 971–1067 of accession X58924) containing the transcriptional terminator of the gene encoding ribosomal protein L11e of *Haloferax volcanii* [13] was PCR amplified with primers that included a 5′ *Hind*III site and a 3′ *Bgl*II site. The primers used were BR1 (5′-TACTCGAAGCTTCTGACGTCTCGGAACCGTCTC-3′) and BR2 (5′-GTGAGATCTTCCGGGTCGAATCGGG-3′) that contain 5′ *HindIII* and *Bgl*II sites, respectively (underlined). The amplified product was digested with *Hind*III and *Bgl*II, ligated to *Hin*dIII + *Bgl*II-digested plasmid, and introduced into *E. coli* cells. A plasmid isolated from ampicillin-resistant transformants was sequenced across the cloning region to confirm the presence, orientation and correct sequence of the terminator. In this way, the L11e terminator was positioned just upstream of the *Nde*I promoter cloning site (the start codon of the *bgaH* ORF), while retaining the *Hind*III site and *Bgl*II sites, and deleting the natural promoter of *bgaH*. The resulting plasmid was designated pRV1. 

The *Nde*I site of pRV1, which overlaps the reporter gene initiation codon (catATG) was replaced by a *Cla*I site (atcgATG; the *Cla*I site is underlined) by insertion of a synthetic linker that replaced the sequence between the *Nde*I and *Afe*I restriction sites of pRV1. The two strands of the linker had the following sequences: 

pRV1 top strand linker: 

5′-GATCTCCACGTTGATCATTGATCGATGACAGTT GGTGTCTGCTATTTCCCGAGCACTGGTCGCGAGAGC-3′; 

pRV1 bottom strand linker:

5′-GCTCTCGCGACCAGTGCTCCGGGAAATAGCAGACACCAACTGTCATCGATC AATGATCAACGTGGA-3′.

The *Cla*I site is underlined in both sequences. The complementary oligonucleotides were annealed, ligated to *Nde*I + *Afe*I digested pRV1, and the products introduced into *E.coli* and transformants grown under ampicillin selection. A colony was selected that contained a plasmid with the expected linker addition, and the resulting 12,135 bp construct was designated as pRV2. A plasmid map is given in Figure S1, and the sequence of pRV2 was deposited under GenBank accession MZ936313.

Primers used to amplify the IRs from halovirus HF2 for ligation into pRV2 are shown in Table S1. These and other promoter amplicons were generated with primers carrying *Bst*B1 (TT′CGAA) tags, which allowed selection against self-ligated plasmids by digestion of ligation mixtures with *Bst*B1. This is because *Bst*B1 and *Cla*I generate complementary overhangs, but ligation of *Bst*B1 and *Cla*1 overhangs destroys both sites. Reporter gene fusions were constructed as follows: pRV2 was digested with *Cla*I and purified (MoBio DNA purification kit; Carlsbad, CA, USA). PCR amplicons were purified, as above, restricted with *Bst*B1, purified again, and then ligated to the vector, as above; 50 ng vector was ligated per reaction. Ligations were performed in a 5 µL total volume, which was diluted to 50 µL for digestion with *Bst*B1 in order to linearize any self-ligated vector, which greatly reduces its ability to transform host cells.

### 2.3. Site-Directed Mutagenesis of Intergenic Repeat IR4

Site-directed mutations were generated using the Invitrogen GeneTailor kit (Invitrogen; Carlsbad, CA, USA), which allows the use of a mutagenic oligonucleotide primer to produce the desired mutation from cloned DNA that has been previously methylated. The procedure also incorporated PCR amplification, and after the introduction of the reaction products into *E. coli* DH5α, unmutated template plasmid was degraded by the *Mcr*BC endonuclease that digested methylated DNA, allowing the efficient recovery of the amplified (mutated) plasmid. IR4 was cloned between the *Hind*III-*Eco*RI sites of pBlueScript SK II+ and used for mutagenesis, after which the mutations were confirmed by sequencing. IR4 variants were then amplified using forward and reverse primers with engineered *Cla*I tags, digested with *Cla*I and ligated to the BgaH reporter plasmid pRV2 (see above).

### 2.4. β-Galactosidase Assay

β-galactosidase assays were performed using a protocol modified from that described in the HaloHandbook [10]. Briefly, cultures of *Hfx*. *volcanii* DS52 strains carrying promoter–reporter plasmids were cultivated in MGM to OD_600_ 0.4–0.7. As a negative control, *Hfx*. *volcanii* DS52 carrying the reporter plasmid without any insert was used. To measure BgaH activity, 50 μL of culture was added to 350 μL BgaH buffer (2.5 M NaCl, 50 mM Tris-HCl pH 7.2, 10 μM MnCl_2_, 0.1% *v/v* 2-mercaptoethanol) and 50 μL 1% *v/v* Triton X-100, 20% *v/v* Tween 20 in a 1.5 mL plastic microcuvette tube. Samples were vortexed briefly prior to the addition of 50 μL ONPG (8 mg/mL in BgaH buffer). Reactions were incubated at 32 °C and the OD_405_ measured at regular intervals until it had reached 0.7–1.0, after which the entire reaction volume was quickly transferred to a plastic tube containing 700 μL of 1 M Na_2_CO_3_ (to stop the reaction), and vortexed briefly.

For each assay, at least four time points were used to generate a curve for each construct, and β-galactosidase specific activities (SA) were calculated according to the equation [ΔOD_405_ ÷ ΔT] × [1000 ÷ (culture volume × OD_600_)], where ΔOD_405_ measures the change in OD_405_ over a time period (ΔT) measured in minutes. ΔOD_405_ was taken from the linear region of the resulting curve. OD_600_ refers to the optical density of the culture at the time of the assay. Assays were completed in duplicate, and 3 biological replicates were used for each plasmid construct.

### 2.5. RT-PCR

Cultures were grown to OD_600_ ~ 0.4 before isolation of total RNA using Trizol (Invitrogen; Carlsbad, CA, USA), as described in Section 2.7. The quality of preparations was determined by electrophoresis on 2% Tris-acetate-EDTA (TAE) gels containing 20 mM (final concentration) guanidine thiocyanate. 

Reverse transcription was at 50 °C for 1 h, using 1 μg of RNA as template with 1 μg specific primer. AMV reverse transcriptase (Promega; Madison, WI, USA) was used according to the manufacturer′s instructions. Following cDNA synthesis, cDNA templates were purified using the Qiaquick PCR clean-up kit (Qiagen; Germantown, MD, USA), with elution in 50 μL pure water. PCR reactions were performed with 25 μL GoTaq mastermix (Promega; Madison, WI, USA) and 5 μL cDNA template according to the manufacturer′s instructions. PCR reactions were run for 35 cycles, with the annealing temperature being equivalent to the lower Tm of the primers for the first 5 cycles. Thereafter, the annealing temperature was 5 °C lower. Primers are listed in Table S2. 

All RT-PCR experiments included a number of controls to exclude false-positive or false-negative results. The following controls were performed alongside test reactions: (1) a positive control consisting of HF2 genomic DNA; (2) a negative control consisting of RNA extracted from uninfected cells, which was mock reverse-transcribed to confirm that cDNAs and PCR products were virus-specific, and not the result of mis-priming on the host transcriptome; (3) a negative control consisting of RNA (harvested from infected cells) that was not reverse-transcribed, in order to show that positive signals were not due to amplification of incompletely digested viral DNA.

### 2.6. Infection of Hrr. coriense with HF2

For infection with virus, cultures of *Hrr. coriense* were grown to OD_600_ ~0.3–0.4 at 37 °C, with orbital shaking (180 rpm). HF2 virus was added (MOI ~ 50) and the cells were incubated without shaking for 30 min (RT) to allow virus absorption, after which the culture was shaken at 110 rpm for 5 h until harvesting for RNA. 

### 2.7. RNA Extraction

RNA was extracted from *Hrr. coriense* cultures 5 h p.i. using Trizol (Invitrogen; Carlsbad, CA, USA). Cells from 5 mL cultures were pelleted (bench top centrifuge, 14,000 rpm) and resuspended in 1 mL Trizol. After incubation (RT, 5 min), 200 μL chloroform was added, the mixture shaken vigorously, and incubation continued (5 min). Mixtures were centrifuged (14,000 rpm, 15 min) to separate aqueous and phenol-chloroform phases. The aqueous phase was removed to a fresh tube, and RNA was precipitated by the addition of 0.5 mL isopropanol. Following incubation (15 min, RT), and pelleting (14,000 rpm, 15 min), the RNA was washed twice with 75% *v/v* ethanol, and the pellet was then air-dried. 

Before use in RT-PCR assays, pellets were resuspended in 50 μL RNase free water. DNA was hydrolysed by the addition of 20 U RNase-free DNase I (New England Biolabs; Ipswich, MA, USA) and incubation at 37 °C for 1 h, after which the enzyme was inactivated by heat treatment (75 °C for 10 min). The concentration of RNA was determined spectrophotometrically, and the quality was assessed by denaturing agarose gel electrophoresis. 

### 2.8. Primer Extension Assays

Primers are listed in Tables S2 and S3, and were designed with a 5′ terminal guanosine residue to maximise labelling efficiency [14]. Primers were radiolabelled with ^32^P-ATP using polynucleotide kinase (New England Biolabs, Ipswich, MA, USA), after which the labelled primer was recovered by ethanol precipitation. Extension reactions used 10 µg RNA as a template, labelled primer and Promega AMV Reverse transcriptase, and followed the manufacturer′s instructions. The resulting cDNA was purified (QIAquick PCR purification kit, Qiagen) and resolved by electrophoresis on a 6% acrylamide urea sequencing gel alongside a sequencing ladder. DNA sequencing ladders were prepared using labelled primer on either HF2 DNA or reporter plasmid pRV2 DNA. In the latter case, the primer Bgal pEXT (5′ GCCATCTGACTGATATCGGTCTCC 3′) was used in PCR with a forward (unlabelled) primer BR1 (5′ TACTCGAAGCTTCTGACGTCTCGGAACCGTCTC 3′), and 1 ng of unlabelled pRV2 plasmid as template. Following PCR, the amplicon was column purified, and the equivalent of 800 counts/s added to 4 µg salmon sperm DNA in a total volume of 14 µL. To produce a purine ladder, 3 µL of 8.8% *v/v* formic acid was added, and the mixture incubated (7 min, 37 °C). Reactions were immediately placed on ice (5 min) prior to the addition of 150 µL 0.1 M piperidine and incubation (at 85 °C, 30 min). Reactions were then precipitated twice (with ethanol), before the pellet was resuspended in 10 µL H_2_O.

Primer extension assays either used RNA templates extracted from HF2-infected *Hrr. coriense* cultures, or RNA extracted from *Hfx. volcanii* carrying pRV2, into which putative HF2 promoters were ligated. Putative promoters were fused at the translation initiation codon of the *bgaH* gene, and primer extension was performed using the primer Bgal pEXT which anneals seventy-three nucleotides downstream of the *bgaH* translation initiation codon.

### 2.9. Phylogenetic Reconstruction

Viral genome sequences were aligned within the Geneious Prime v2021.2.2 (Auckland, New Zealand) environment using the MAFFT aligner tool and trees inferred using the Geneious Tree Builder option with the genetic distance model of Tamura-Nei, the Neighbor-Joining algorithm and 100 bootstrap replications.

## 3. Results

### 3.1. Intergenic Repeats of HF2

**Figure 1:**
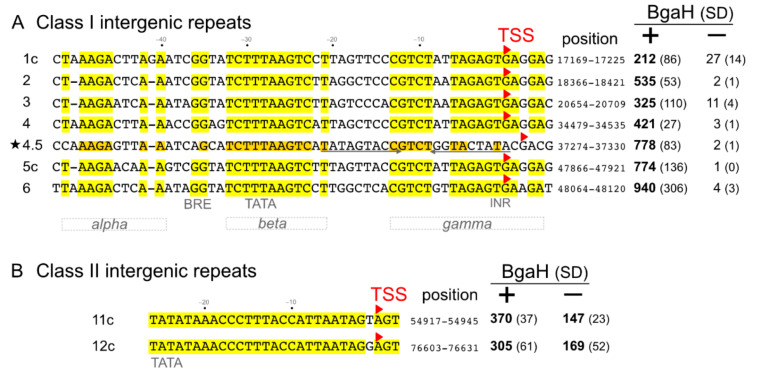
Intergenic repeats of HF2, transcription start sites (TSS) and promoter activity. Class I IRs are shown in panel (**A**), and class II IRs are displayed in panel (**B**). The aligned IRs reported by [3] are shown with matching bases highlighted in yellow, except for IR4.5 (asterisked), which uses orange highlighting (see text). The suffix ‘c′ after the IR number (e.g., 1c) indicates that the sequence shown is from complementary strand of HF2 according to the sequence accession AF222060.2. Conserved motifs (*alpha*, BRE, TATA, *beta*, *gamma* and INR) are described in the text. The base positions are given at the right. Transcription start sites are indicated by red arrowheads above the initiating base. Promoter activity in the natural orientation (+) and reverse orientation (−) were measured using the reporter plasmid pRV2 (see Methods), and specific activity values of BgaH are given along with the standard deviation (SD).

The IRs of halovirus HF2 (AF222060.2) were discovered and described in a previous study [3]. Figure 1 shows alignments of the two classes of IRs, with identical bases highlighted. An additional class I repeat (4.5, asterisked) was detected in the present study. It is less similar to the other six class I repeats, and the bases that it shares with others are shaded orange. 

The seven class I repeats display blocks of identical bases interspersed with more variable positions. The three most conserved motifs within class I repeats were designated *alpha*, *beta* and *gamma*, and these are indicated at the base of the alignment in Figure 1A. The 12 nt *beta* sequence is only found in IRs and does not occur elsewhere in the genome. Even shorter samplings of this motif, such as TCTTTAAGTC (10 nt) or CTTTAAGT (8 nt) remain specific for these 7 IRs. The 19 bp *gamma* sequence (excluding IR4.5) is also only found in class I IRs, as is the central 12 bp sequence CGTCTNNTAGAG. The *gamma* motif is absent in IR4.5 and is replaced by a 9 bp inverted repeat (underlined in Figure 1A). The only other occurrence of this inverted repeat is at nt 17135–17155, located 15 bp downstream of IR1c. 

The two class II repeats are 29 bp long, near identical in sequence, and contain three AT motifs (4, 7 and 8 bp in length), each separated by two or three C residues. These class II IR sequences do not occur elsewhere in the genome and are so strongly distinctive that even an 8 bp substring CCCTTTAC taken from the middle of the consensus is only found in these two IRs.

### 3.2. Promoter Activity of Intergenic Repeats

All class I and class II IRs were cloned into the reporter plasmid pRV2 (Figure S1) immediately upstream of the *bgaH* gene. Both orientations of each IR were tested, and promoter activity was measured by enzyme assays of beta-galactosidase BgaH (Figure 1). All IRs showed a strong promoter activity in their native orientation (as shown in Figure 1). In the reverse orientation, class I IRs showed low (IR1c, IR3) or negligible activity, while class II IRs displayed about half the activities compared to those of their native orientation.

The transcription start sites (TSS) of the IRs in their native orientation were then determined by primer extension, and the initiating bases are indicated by red arrowheads in Figure 1. Within the class I repeats, all but IR4.5 initiate at the same G within the *gamma* motif. IR4.5 is poorly conserved in this region and has a TSS at a G two bases further in the 3′ direction. From these results, the TATA box is deduced to be the AT-motif beginning at −26 from the TSS, and the BRE is just 5′ of this (−33 to −37), which is in remarkable agreement with promoter studies in model haloarchaea such as *Hfx. volcanii* [15,16]. The TSS of IR4.5 has an offset of two bases compared to the other class I IRs. Both class II IRs initiate at an A, and the likely TATA box is within the 8 bp AT-motif at −19 to −26.

### 3.3. Scanning Mutagenesis of an Intergenic Repeat

**Figure 2:**
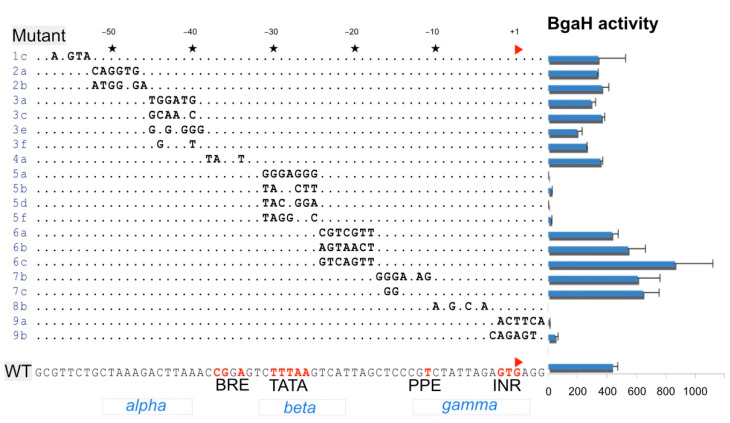
Scanning mutagenesis of IR4 (nt 34479–34535) and the effect on its promoter strength. The sequence of HF2 IR4 is shown at the bottom of the figure (WT), with bases conforming to promoter consensus motifs coloured red and labelled underneath (BRE, TATA, PPE and INR; see text). Below these, the conserved class I IR motifs (*alpha*, *beta*, *gamma*) are given (see Figure 1). The initiating base (TSS) is indicated by a red arrowhead at +1. Mutants of IR4 are labelled at the left side and bases that differ from wt are shown, while unchanged bases are indicated by dots. At the right, the promoter strength (BgaH activity) of mutant and wt IR4 sequences are presented in bar chart format, with bar heights showing BgaH enzyme specific activities (see Methods). The scale is given below in SA units (see Methods). Whiskers indicate one standard deviation from the mean.

The effects of mutations along the length of IR4 on promoter activity were investigated by scanning mutagenesis, and the results are shown in Figure 2. Promoter activity was severely diminished by mutations of the TATA box and around the transcription initiation region (INR). Alterations to the B responsive element (BRE) and the conserved class I region *alpha* did not affect promoter activity. Changes in the *beta* region outside the TATA box (mutants 6a, 6b and 6c) had no negative impact on promoter activity. Mutant 8b alters the *gamma* conserved region and completely abolished activity.

### 3.4. Transcripts Identified by RT-PCR

The previously published transcription map of HF2 used Northern blot hybridisation and did not detect transcripts longer than 9 kb [3], yet much longer transcripts could be expected if all successive and closely spaced CDS were encoded on the same transcript. To search for longer transcripts, an RT-PCR approach was chosen that was previously shown to detect transcripts of up to 15 kb in length in cells infected by the sphaerolipovirus SH1 [17]. 

**Figure 3:**
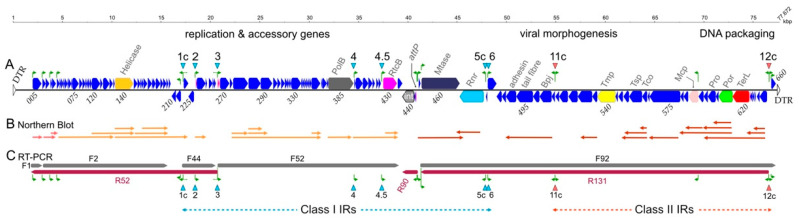
Transcript maps of HF2 by RT-PCR (**C**) and Northern blot hybridisation (**B**). The HF2 gene map is shown at the top (**A**), with gene orientation indicated by arrow direction and position above or below the line. IRs are labelled and indicated by downward pointing arrowheads: blue for class I IRs and red for class II IRs. They are also repeated below panel C. Mapped transcription start sites (TSS) and their directions are indicated in (**A**) by green arrows above the genes (see text for details) and are also shown underneath the transcripts in (**C**). Transcripts in panel (**C**) are coloured according to orientation: grey (forward) and crimson (reverse).

HF2 transcripts produced at 5 h p.i. (late in the infection cycle) were analysed by RT-PCR and the results are presented in Figure 3, and an example of the data is given in Figure S2. For comparison, the Northern blot data of Tang et al. [3] are shown immediately above these.

Eight specific transcripts were detected by RT-PCR, ranging in size from ~1 kb to ~35 kb, with the largest covering 41 ORFs (Figure 3, Table S4). Each transcript was designated by its direction (forward or reverse) and a number, and is coloured grey (forward) or crimson (reverse) in Figure 3. For example, F1 is a short, forward transcript beginning at the first annotated CDS. Together, these transcripts cover the entire predicted coding region of the genome, with about 70% being transcribed from both strands. Reverse-strand transcription was not detected across the 9.7 kb region from 20.7–30.4 kb. In general, the coding transcripts encompassed the gene modules present on each arm of the genome, with the two longest modules on the left arm transcribed as two, inwardly directed (sense) transcripts (F2 and F52) while the virus morphogenesis module of the right arm was transcribed (in the sense direction) as one single, long transcript (R131).

Two IRs are found at the 5′ ends of long transcripts. The class I IR3 directs the synthesis of an 18 kb transcript (F52) that includes genes for PolB and RtcB. The class II IR12c directs a 35 kb late transcript (R131), which spans the virus morphogenesis genes. The promoters of these IRs show activity in the opposite direction (Figure 1) and could direct the transcription of transcript R52 (IR3), and the last CDS (HrrHF2_660; IR12c). 

Three IRs (IR4, IR4.5 and IR11c) are close to the 5′ ends of transcripts mapped previously by Northern blot hybridisation. However, not all of the major transcripts can be explained by the IR-associated promoters, such as transcripts from the left end of the genome, or those emanating from the 41 kb region.

### 3.5. Transcripts Initiating from Promoters Outside of IRs

**Figure 4:**
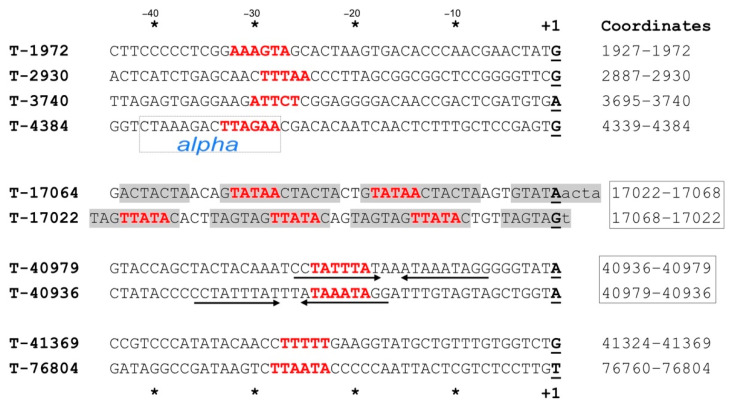
Transcription start sites (TSS) that are not within IRs. TSS were determined by primer extension (see Methods) and are shown as bolded, underlined bases at the right edge, and labelled above and below as +1. Coordinates for the bases displayed are given at the right edge. Boxed coordinates indicate complementary regions of the genome, where outwardly directed TSSs occur either side of the same (or closely spaced) promoter. Grey shading in T-17064 and T-17022 denotes repeat motifs. Potential TATA boxes are in bold, red font. Multiple TATA boxes are shown in T-17064 and T-17022 because of the repeat motif, but only those at −28 (T-17064) and −24 (T-17022) have the appropriate spacing. Asterisks at top and the bottom indicate 10 bp intervals upstream of the TSS (+1). Arrows underlining sequences in T-40979 and T-40936 denote inverted repeats. Boxed region of T-4384 sequence is identical to the *alpha* class I IR conserved region of IR1c (see Figure 1 and Text).

The start sites of transcripts not initiating at IRs were located by primer extension (Figure 4) and are shown as green arrows in the genome map of HF2 (Figure 3). Three TSS were found in the region around 41 kb (T-40979, T-40936 and T-41369), and were consistent with transcripts proceeding outwards in both directions from this part of the genome. One of these (T-41369) is the TSS for the long methyltransferase gene (HrrHF2_460).

A 14 bp sequence (CTAAAGACTTAGAA) overlapping the promoter upstream of TSS T-4384 (boxed in Figure 4) is identical to the *alpha* region of IR1c (Figure 1), and closely similar to the other class I IRs, suggesting that this represents a common regulatory motif. The promoters for TSS T-17064 and T-17022 are embedded in regularly spaced, direct repeats (grey shading in Figure 4); two perfect 12 bp repeats flanked by two imperfect versions of the same repeat. The spacing between repeats is 14–15 bp, or about 1.5 helical turns of DNA. 

### 3.6. tRNA-like Gene HrrHF2_450 of HF2 Is attP

The HF2 genome carries a site-specific integrase gene (HrrHF2_440) nearby a tRNA-like gene (HrrHF2_450), a typical configuration found in temperate viruses that can integrate into their host genome [18]. The predicted tRNA has a low confidence by tRNA-scan (infernal score 28.2) and does not fold into a conventional secondary structure. Related viruses (Hardycor2, Serpecor1) have corresponding tRNA-like genes that are truncated by 15 nt at their 5′ ends (HrrHc2_210, HrrSp1_205), reducing their length to 57 nt and rendering them non-functional. The HF2 tRNA-like gene HrrHF2_450 most likely functions as an *attP* sequence, allowing recombination into a host tRNA gene, but HF2 is strictly lytic on *Hrr. coriense*. Examination of the *Hrr. coriense* genome shows it does not carry a tRNA gene matching the sequence of HrrHF2_450, explaining why HF2 is virulent on *Hrr. coriense*. Certain species of *Halorubrum* carry tRNA genes exactly matching the terminal 57 nt of the HF2 *attP*, i.e., *Hrr. trapanicum* strain CBA1232 (AP017569.1) and *Hrr. sodomense* strain MBLA0099 (CP073695.1), making it likely that entry of the HF2 genome into cells of these strains would allow site-specific integration. Two of the three remaining annotated tRNA genes of HF2 (tRNA-Arg, HrrHF2_230; and tRNA-Asn, HrrHF2_260) show high predictive scores and good inferred secondary structures by both tRNA-scan and Aragorn algorithms [19,20], while the third tRNA (tRNA-Pro, HrrHF2_625) has both a weak predictive score and an atypical inferred secondary structure (data not shown), and its function is uncertain.

### 3.7. Conservation of IRs and a Complex Bidirectional Promoter Region in Haloferacalesviruses

**Figure 5:**
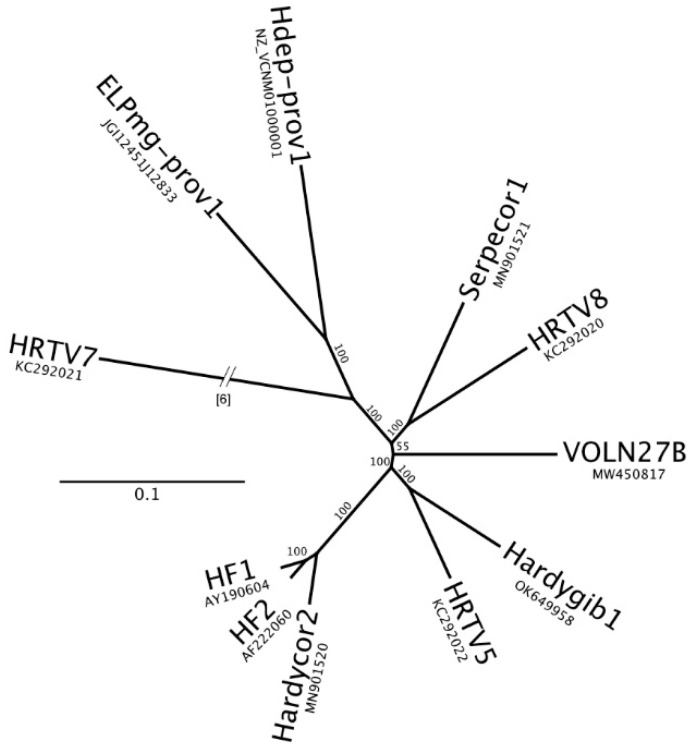
Inferred phylogeny of haloferacalesvirus isolates and two related proviruses (ELPmg-prov1 and Hdep-prov1 [6]), based on whole genome sequences. After MAFFT alignment there were 21,264 parsimony informative sites. Scale bar, 0.1 expected changes per site. The branch for the outgroup (HRTV7) was truncated and the length shown at the indicated break. Bootstrap confidence values are shown at branchpoints. Sequence accessions are given below each virus name.

A genome-based phylogenetic tree reconstruction of *Haloferacalesvirus* (Figure 5) shows the relationships between the current virus isolates belonging to this genus, and two related provirus sequences [6]. HF2 is most closely related to HF1 and Hardycor2, and these form a sister clade to HRTV5 and Hardygib1. The details regarding the isolation and genome sequence of Hardygib1 (accession OK649958) are given in the Supplementary Materials (Text S1).

If IRs are important transcription and regulatory control points of the viral genome, then they might be expected to be conserved in position and orientation among related viruses. The genomes of haloferacalesviruses were aligned and the results (Figures S3 and S4) confirmed that both classes of IR were maintained in corresponding positions (and orientations) and showed strong conservation in sequence compared to HF2. The two class II IRs (Figure S4) show almost complete conservation across all the other haloferacalesviruses, with only IR11c showing a G to T change in four cases at the same position (just downstream of the TSS). The class I IRs are more variable, but the individual motifs are often highly conserved. For example, the TATA box containing *beta* motifs of the other viruses differ by only three base changes from HF2 (Figure S3). The *alpha* motif of IR6 shows almost complete sequence conservation while it is less conserved in the other IRs. The *gamma* motif (that includes the TSS) shows the greatest conservation from 7 nt upstream of the TSS to 1 nt downstream, and in IR1c, the *gamma* motif is identical in all viruses. 

The complex, bidirectional promoter region between nt 17022–17068 (Figure 4) that contains repeats motifs, was also seen to be strongly conserved across haloferacalesviruses (Figure S5). Divergence parallels the inferred viral phylogeny (Figure 5). In six viruses, the three 12 bp direct repeat motifs (boxes 1–3) retained a 15 bp spacing, while three had a 2 bp deletion that removed the bases separating box1 and box2. The deletion was even more pronounced in ELPmg-prov1, where the box2 repeat was lost. A curious feature of the first major repeat (box 1) was the change from CT to AG in four viruses (Serpecor1, HRTV8, Hdep-prov1 and ELPmg-prov1), making it identical to the box 3 repeat of most viruses (Figure S5).

## 4. Discussion

The class I and class II intergenic repeats, which were previously identified in the genome sequence of HF2 and thought to contain promoter motifs [3]**,** were demonstrated to contain viral promoters. Class I IRs are found between position 16 kb to 49 kb of the HF2 genome and are involved in the transcription of early-middle genes; while class II IRs are found in the right arm between 49 kb and the right end and likely regulate the late gene transcription of virus morphogenesis genes. Both classes of IRs are well conserved in the genomes of related viruses, supporting their functional significance. The distinct difference in the consensus sequence of the two IR classes suggest a regulatory model, where sequence-specific, virus-encoded factors interact with IRs to control viral gene expression in a programmed and temporal manner. Since the promoters of individual IRs were found to be active in the cells of *Hfx. volcanii,* where other HF2 genes were not present, virus-encoded factors were not required for their activity. The potential role of host cell factors in regulating the promoter activity of IRs needs to be determined. The transcription of genes located between the left end and 16 kb utilize promoters that do not show any extended sequence pattern and are probably unregulated. HF2 does not encode an RNA polymerase to drive virus-specific gene expression in the same way that bacterial viruses, such as T7, do [5]; therefore, this is dependent on the host enzyme. This is similar to lactococcal *Siphoviridae*, belonging to the 936 group [21], which have linear dsDNA genomes with genes arranged in modules that face inwards, with one arm carrying early genes and the other arm carrying late-expressed genes involved in virus morphogenesis. The temporal regulation of HF2 middle and late gene transcription is likely governed by virus-encoded proteins that influence when and how the host RNA polymerase interacts with IRs and the promoters they contain. In the first 16 kb of the genome, which is transcribed first, there are no IRs but many candidate genes that could specify IR-regulatory proteins, such as those specifying proteins with CxxC motifs indicative of zinc-finger domains [22], one of which (HrrHF2_165) also carries a predicted helix-turn-helix domain [23].

Scanning mutagenesis of IR4 provided more detailed insight into the important promoter elements contained within a representative member of the class I intergenic repeats. Alterations to the TATA box and the sequence around the TSS greatly reduced the measured promoter activity, which was consistent with earlier studies that identified these conserved promoter elements in haloarchaea using bioinformatics [15,16,24], and by in vivo studies [25,26]. It is likely that IRs contain binding sites for viral regulatory proteins, but to examine this in detail will require more advanced experimental systems. 

Surprisingly, class II IRs display a high promoter activity in both directions when tested using the BgaH reporter plasmid in *Hfx. volcanii*; their activities in the reverse direction being about half of those in the forward direction. In the case of IR12c, this is consistent with its location at a point where the coding strand changes, allowing it to drive the outward transcription of the CDSs found on either strand of the genome. Two of the class I IRs (IR1c and IR3) also show a promoter activity in the reverse direction in the same reporter system, although much weaker than in the forward direction. In the case of IR3, this initiates two outwardly directed and long transcripts (R52 and F52). How these dual promoters function within the confines of IRs without interfering with each other remains to be examined. Interference between closely spaced, outwardly facing promoters was previously reported in phiH1 [27], a temperate myovirus infecting *Halobacterium salinarum* [27,28,29].

Long viral transcripts were detected by RT-PCR in HF2 infected cells. These encompassed well-defined gene modules where genes were closely spaced (or overlapping) and in the same orientation, such as the 35 kb transcript across the virus morphogenesis module. However, the previous Northern blot study of HF2 revealed transcripts that ranged in size from 0.5–9 kb, with most being between 1–2.5 kb [3]. One explanation for these differing results is that long primary transcripts were processed into smaller species. Such processing was well described in phiH1 [29,30,31] which synthesized long primary transcripts, one of which was at least 20 kb, that were subsequently processed to smaller RNAs. The synthesis of long viral transcripts is not limited to caudoviruses, as long sense and anti-sense transcripts were previously reported for SH1, an alphasphaerolipovirus, using the RT-PCR approach [17,32]. 

Long anti-sense counter transcripts were a significant observation in the present study, and they are likely to function in regulating viral gene expression. Anti-sense RNAs are well known in bacteria and their viruses, such as lambda [33]. The first example in Archaea was found in halovirus phiH1 [34], which synthesizes a counter transcript (T_ant_) complementary to an early lytic transcript (T1). These bind to produce an RNA hybrid that becomes a substrate for an endogenous RNase, rendering the mRNA untranslatable. This processing was later shown to be catalyzed by a structure-specific RNase that attacks the ends of RNA duplexes [35]. Recent RNA-seq studies [16,36] in *Hfx. volcanii* also demonstrated that a high level of counter-transcription (‘cis-antisense′) occurs in haloarchaea, producing many transcripts complementary to mRNAs.

The potential temperate state of HF2 and other haloferacalesviruses, and the regulation of the lytic–lysogenic switch, remain unexplored. Once the genes involved in decision-making and lysogeny are identified their functions can be analysed in detail and compared to well-studied temperate bacteriophages such as lambda [37]. Although HF2 carries an integrase gene nearby a putative *attP* sequence, the host species used (*Hrr. coriense*) does not possess a homologous tRNA for integration, explaining why infection is lytic. The three-gene module consisting of *attP*, a small CxxC domain protein, and integrase (HrrHF2_450, 445 and 440) is situated between the middle- and late-expressed regions and found to be transcribed from a single promoter (T-40936) that is not part of an IR. An added level of complexity is that the TATA-box of this promoter also functions in the opposite direction (T-40979), across genes for a small hypothetical protein (HrrHF2_455) and N-6 DNA methylase (HrrHF2_460). 

Hdep-prov1 provides a natural example of the integrated state of a haloferacalesvirus and is found in the genome of *Hrr. depositum* Y78 integrated at a tRNA gene [6]. Its recombination into the chromosome would have required the circularization of the viral genome prior to site-specific integration. Only one copy of the direct terminal repeat (DTR) is found in Hdep-prov1, suggesting that the termini were joined by homologous recombination. However, in the related halovirus HF1, the termini appear to be cohesive, presumably by single-stranded DNA overhangs [7]. The presence of DTRs on the HF2 genome resembles coliphage T7-like viruses and, although T7 does not carry an integrase, there are T7-like cyanoviruses such as P-SSP7 that do, and are thought to be capable of integration [38]. They may provide useful models for comparison.

Much remains to be discovered but a continuing obstacle is the lack of genetically tractable and robust host strains in which the genes of these viruses can be analysed in detail. However, in other halovirus groups, such as the recently described siphovirus HFTV1 (host *Hfx. gibbonsii* LR2-5) this aspect looks promising. Nonetheless, virus–host systems to study lysogeny in haloferacalesviruses may be best studied by engineering a model host strain, such as *Hfx*. *volcanii*, to carry the homologous tRNA gene and express the cognate cell surface receptor. The latter strategy would also enable the functions of all genes to be studied using the full range of genetic tools.

## Data Availability

The sequence of plasmid pRV2 is available at Genbank under the accession MZ936313. The genome sequence of halovirus Hardygib1 has the accession OK649958.

## Supplementary Materials

The following are available online at https://www.mdpi.com/article/10.3390/v13122388/s1, Figure S1: Reporter plasmid pRV2, Figure S2: Example of RT-PCR results, Figure S3: Conservation of class I Intergenic Repeat regions within members of *Haloferacalesvirus*, Figure S4: Conservation of class II Intergenic Repeat regions within members of *Haloferacalesvirus*, Figure S5: Conservation of T-17064 and T-17022 promoter region within members of *Haloferacalesvirus*, Table S1: Primers used to amplify IRs for ligation to the reporter plasmid, Table S2A: HF2 oligonucleotide primers used for RT-PCR or primer extension reactions, Table S2B: Oligonucleotide primer used for primer extension on transcripts generated by reporter plasmid pRV2, Table S3: HF2 infected cell transcripts detected by RT-PCR, Supplementary Text S1: *Haloferacalesvirus* isolate Hardygib1.
