# Supplementary material for: Halovirus HF2 Intergenic Repeat Sequences Carry Promoters
Source: Viruses. 2021 Nov 29;13(12):2388. doi: 10.3390/v13122388 (PMC8707807; doi:10.3390/v13122388)
Supplement: Supplementary file 1 [file viruses-13-02388-s001.zip › viruses-1477057-supplementary.pdf]

Supplementary Figure S1.

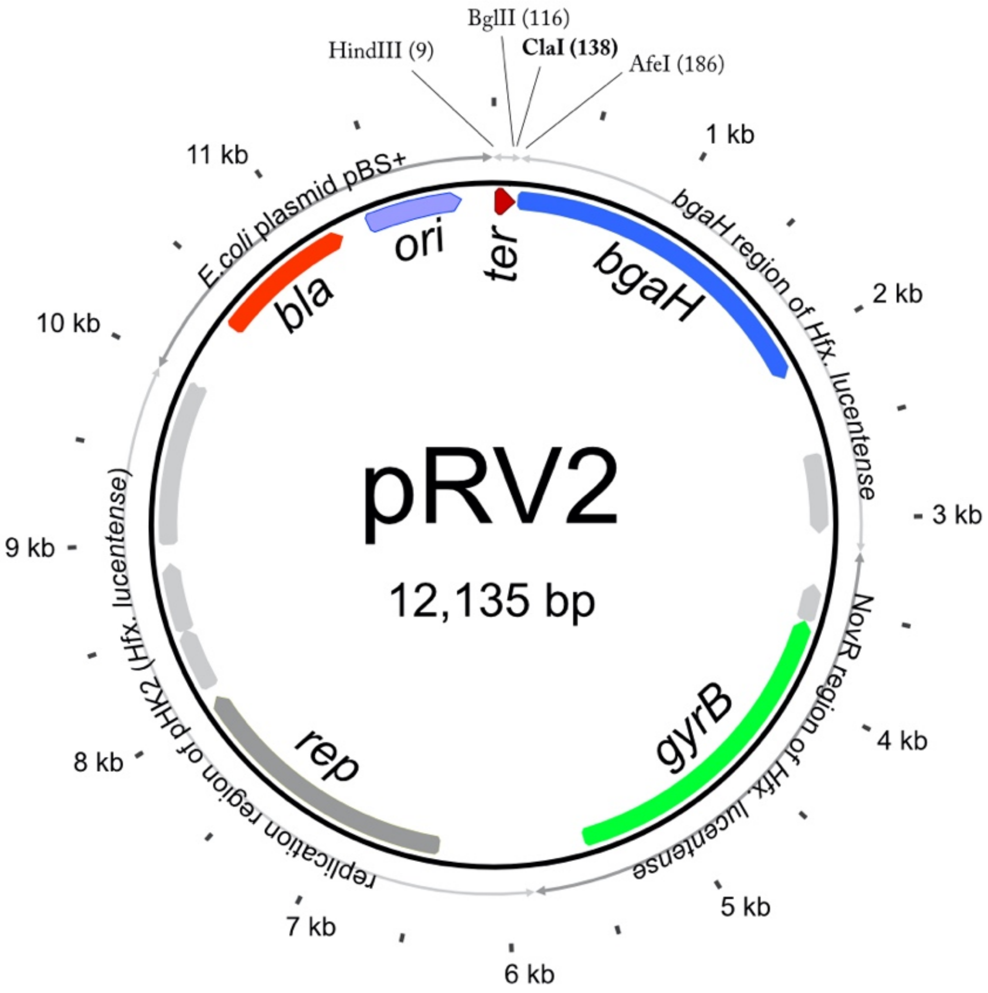

Figure S1. Map of reporter plasmid pRV2 (see Methods for construction details). The circular plasmid is 12,135 bp in length (accession MZ936313), and a scale is given around the outer perimeter (in kb). The next inner level shows the sources of sections of the plasmid, which are indicated by labelled, arrow-headed, grey arcs. Genes are depicted on the innermost level, and are represented as arrows with the most significant being labelled; *bla*, beta-lactamase; *ori*, *E.coli* replication origin; *ter*, L11e transcription termination sequence from *Hfx. volcanii*; *bgaH*, beta-galactosidase from *Hfx. lucentense*; *gyrB*, DNA gyraseB (NovR) from a *Hfx. lucentense* novobiocin resistant mutant; and *rep*, replicase from plasmid pHK2 of *Hfx. lucentense*. The *Cla*I site at the start codon of *bgaH* is used for insertion of sequences to be tested for promoter activity.

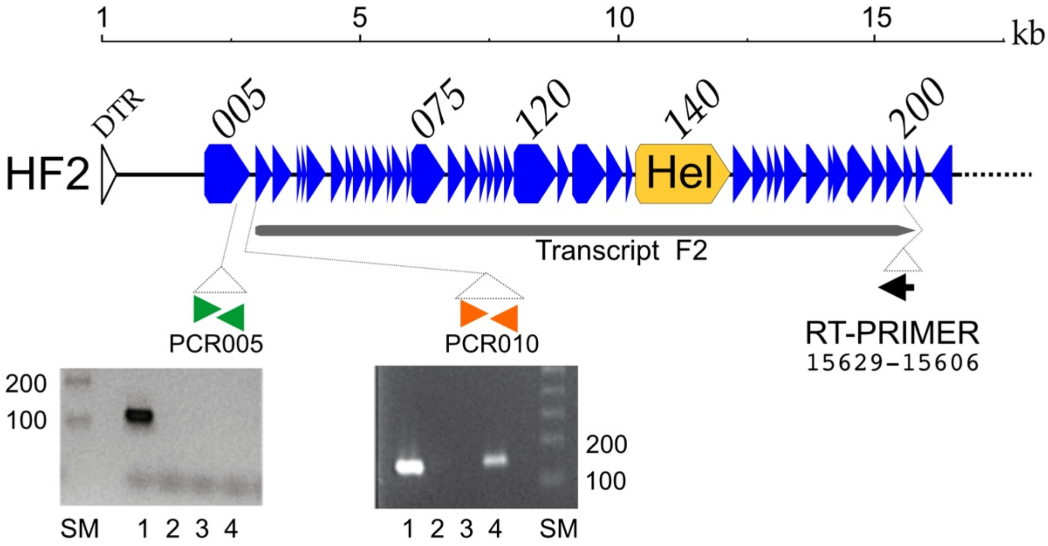

Figure S2. Example of RT-PCR results**.** At the top is a gene map of the left end of the HF2 genome with a scale bar (kb) above. DTR, direct terminal repeat. Numbers 005 - 200 refer to locus tag numbers of the CDS (blue arrows). Below this is a grey arrow representing transcript (F2) that spans HrrHF2_005 to HrrHF2_200 and was determined in the present study. A cDNA copy of this transcript was first synthesised using the primer 15629-15606 which is complementary to a sequence within the CDS of HrrHF2_200. The length of this cDNA was then determined by PCRs targeting regions that progressively moved towards the 3' end of the cDNA. In this example, only the last two sets of PCR primers are presented (orange arrows for HrrHF2_010 and green arrows for HrrHF2_005), and the results of the PCRs using these primer pairs are shown directly below. PCR products were separated by agarose gel electrophoresis with lane loadings as follows: 1, positive control (HF2 DNA as template); 2, negative control (RNA template); 3, negative control (uninfected cell RNA, mock reverse transcribed), and 4, the cDNA preparation being tested. The same DNA size marker (SM; 1 kb ladder) was used for both gels, with the 100bp and 200bp bands indicated at the side. PCR010 was positive for the presence of cDNA, while PCR005 was negative. This method has previously been described for analysing the transcripts of halovirus SH1 [1].

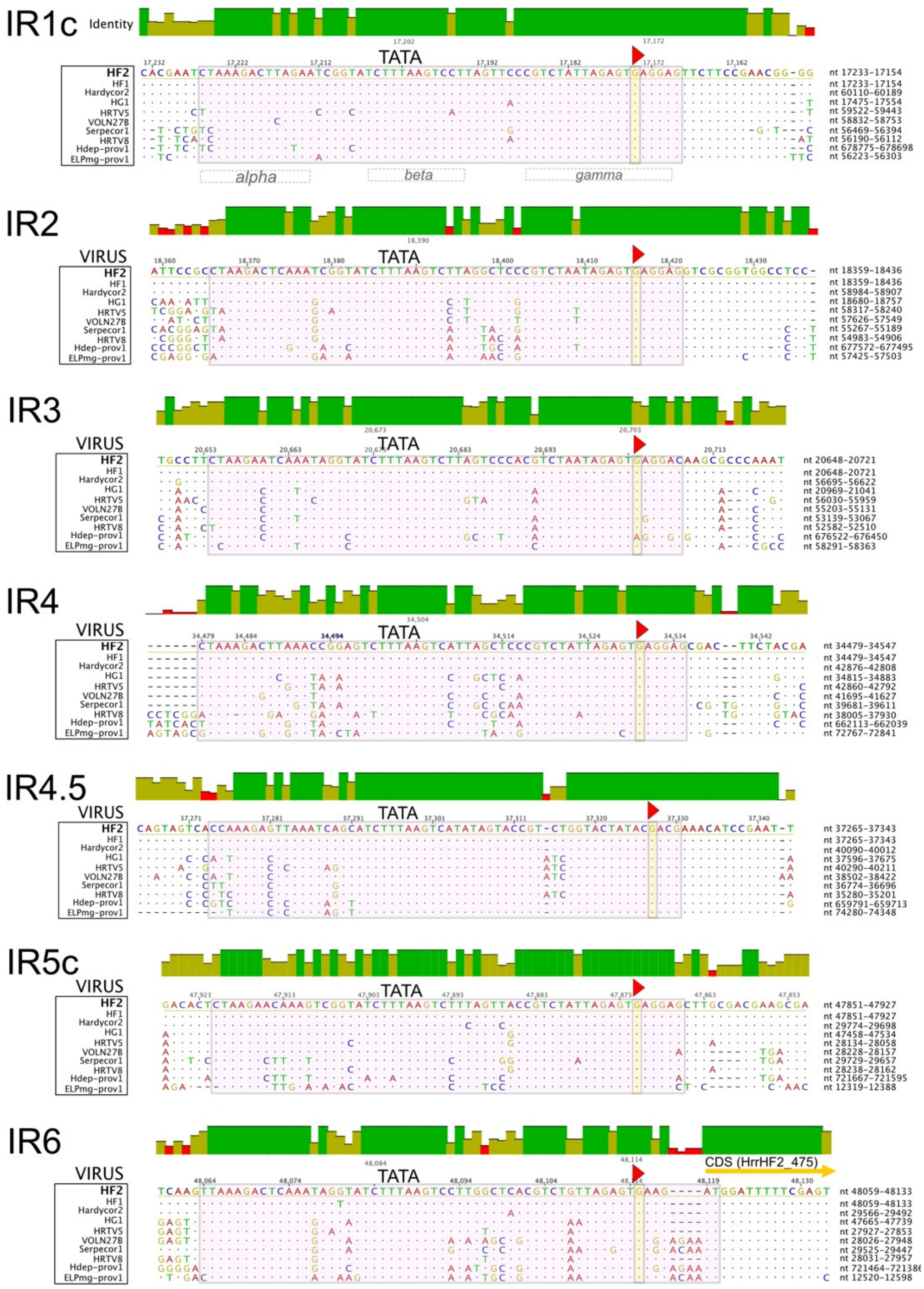

Figure S3. Conservation of class I Intergenic Repeat regions within members of Haloferacalesvirus a. a The IR number of each alignment, and the virus genomes represented on each row of the alignment are shown at the left of each panel. HF2 is the reference sequence and identical bases in the alignment are indicated by dots. Conserved motifs (*alpha*, *beta* and *gamma*) of the IR are indicated under the first panel (IR1c). Pink shading indicates extent of the IR. The TATA box region is indicated above the HF2 sequence, as is the TSS (red triangular arrow). Above each panel is a graph of base similarity, with dark green indicating complete identity and red indicating the weakest similarity. Base coordinates are given at the right side of each alignment and correspond to the GenBank accessions for each virus genome, as shown in Figure 5 of the main text. For the two proviruses, the base coordinates are for the contigs or scaffolds that they are found on; contig NZ_VCNM01000001.1 for Hdep-prov1, and scaffold JGI12451J12833_1000001 for ELPmg-prov1 (available from the JGI IMG/M website at https://img.jgi.doe.gov).

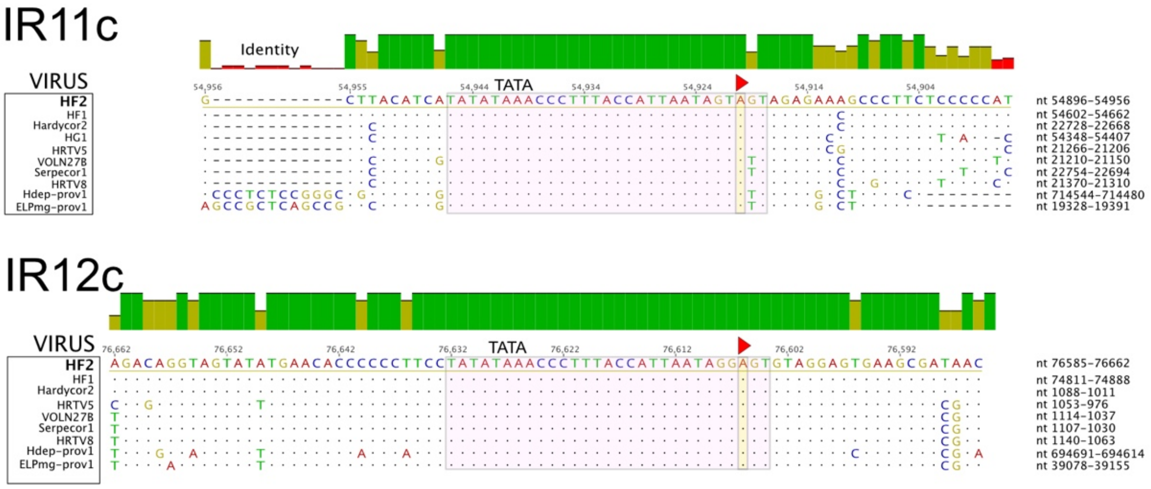

Figure S4. Conservation of class II Intergenic Repeat regions within members of *Haloferacalesvirus* [a]. [a] See legend for Figure S3.

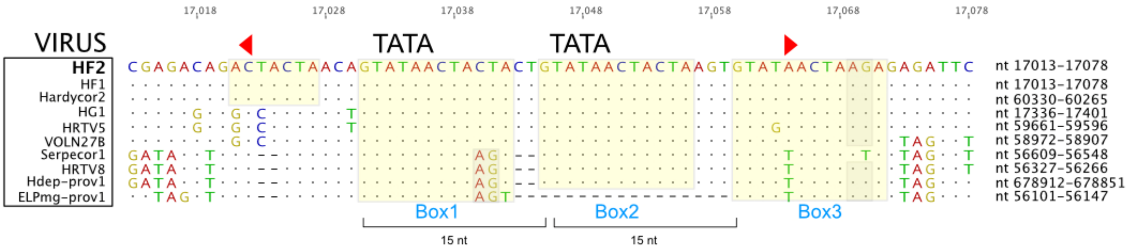

Figure S5. Conservation of T-17064 and T-17022 promoter region within members of *Haloferacalesvirus* [a]. [a] See legend to Figure S3. Repeat sequences are shaded yellow and labeled Box1 to Box3.

Table S1. Primers used to amplify IRs for ligation to the reporter vector pRV2.

Primer	Sequence (5′-3′)[a]	HF2 Coordinates[b]
IRA BSTF	GCGGCGTTCGAAGCAAAGACTCATCTG	2880-2894
IRA BSTR	GCGGCGTTCGAAGGGGAGTCGAACCC	2937-2924
IRB BSTF	GCGGCGTTCGAAGTAAAGACTTAATAC	3666-3681
IRB BSTR	GCGGCGTTCGAACCTCACTCTAAGAG	3706-3693
IRCc BSTF	GCGGCGTTCGAACTAAAGACTTAGAACG	4343-4358
IRCc BSTR	GCGGCGTTCGAACCACTCGGAGCAAAG	4385-4371
IRDc BSTF	GCGGCGTTCGAAGGATGGCGGCGCGG	7941-7954
IRDc BSTR	GCGGCGTTCGAAAGTTGGAACGCTCAT	8001-7986
IRE BSTF	GCGGCGTTCGAAGTATAACTACTACTG	17030-17045
IRE BSTR	GCGGCGTTCGAAAGTTATACACTTAG	17067-17054
IRFc BSTF	GCGGCGTTCGAACTAAAGACTTAGAATC	17226-17211
IRFc BSTR	GCGGCGTTCGAACCTCACTCTAATAG	17171-17184
IRG BSTF	GCGGCGTTCGAACTAAGACTCAAATC	18366-18379
IRG BSTR	GCGGCGTTCGAACTCCTCACTCTATTAGACGG	18421-18402
IRH BSTF	GCGGCGTTCGAACTAAGAATCAAATAG	20654-20668
IRH BSTR	GCGGCGTTCGAACCTCACTCTATTAGACGTGG	20707-20688
IRI BSTF	GCGGCGTTCGAAGCGTTCTGCTAAAGAC	34471-34486
IRI BSTR	GCGCGCTTCGAAACCTCACTCTAATAGAC	34533-34518
IR4J BSTF	GCGGCGTTCGAACCAAAGAGTTAAATC	37273-37288
IR4J BSTR	GCGGCGTTCGAACGTCGTATAGTACCAG	37330-37315
IRKc BSTF	GCGGCGTTCGAAGTGGTAAAACTCAAAGTAGG	37444-37420
IRKc BSTR	GCGGCGTTCGAAAACGCGGGCGAAATATC	37361-37377
IRL BSTF	GCGGCGTTCGAACCTATTTATAAATAA	40953-40967
IRL BSTR	GCGGCGTTCGAAGTCCACAGACGGCATACGG	41001-40983
IRMc BSTF	GCGGCGTTCGAACTCCTCACTCTAATAG	47866-47881
IRMc BSTR	GCGGCGTTCGAACTAAGAACAAAGTCG	47921-47907
IRN BSTF	GCGGCGTTCGAATTAAAGACTCAAATAGG	48064-48080
IRN BSTR	GCGGCGTTCGAACTTCACTCTAACAG	48118-48105
IRPc BSTF	GCGGCGTTCGAACTAAAGACTTAATC	69431-69418
IRPc BSTR	GCGGCGTTCGAAGGTTAACCATAAAG	69390-69403
CL2 IROCII BSTF	**CGAA**TATATAAACCCTTTACCATTAATAGTAGT**TT**	54946-54917
CL2 IROCII BSTR	**CGAA**ACTACTATTAATGGTAAAGGGTTTATATA**TT**	54917-54946
CL2 IRQCII BSTF	**CGAA**TATATAAACCCTTTACCATTAATAGGAGT**TT**	76630-76602
CL2 IRQCII BSTR	**CGAA**ACTCCTATTAATGGTAAAGGGTTTATATA**TT**	76602-76630

[a]Engineered *Bst*B1 tags are underlined. Class 2 IRs were synthesised as linkers with *Bst*B1 overhangs (shown in bold). [b]numbering according to accession AF222060.2.

Table S2. HF2 oligonucleotide primers used for RT-PCR or primer extension reactions.

Primer[a]	Sequence (5'-3')
1712-1727	GCGGCGTTCGAAGGCCTAAAGGGATGTG
1855-1868	GCGGCGTTCGAACTCGCTAAGCAAAC
1926-1913	ATAGCGTTCGAAACCAAACCCCCCAT
2028-2015	ATGGCGTTCGAACCGATGGTGTTCAC
2098-2077	GTGAGCTAAGCTCACGGTGGAG
2880-2894	GCGGCGTTCGAAGCAAAGACTCATCTG
2937-2924	GCGGCGTTCGAAGGGGAGTCGAACCC
3072-3051	GCCTCTGGTCGATTTTGAGGTC
3073-3093	ATCGAGCCTTCGGAGCGATAG
3220-3201	CCTTCGTATTCCTCCGGCTC
3666-3681	GCGGCGTTCGAAGTAAAGACTTAATAC
3706-3693	GCGGCGTTCGAACCTCACTCTAAGAG
3758-3780	ATGTGCCACTTCAACGACGACTG
3837-3817	GAAGCTCCTCCTACGTGGGTC
3881-3860	GGCAGTCTGAGTATCCGCAAGC
4018-4001	GTCTCGTAGCTCGGTATG
4220-4241	GACAGTAACCGACGCCGACTAC
4343-4358	GCGGCGTTCGAACTAAAGACTTAGAACG
4385-4371	GCGGCGTTCGAACCACTCGGAGCAAAG
4509-4532	TTGAAGGTCGGAGATTACCTCCAC
4606-4585	GTCGGCTCAATCGCAATCGAAC
6436-6418	GTTGTGGATTCCCTCTCCG
6692-6706	GCGGCGATCGATATGAGTGTTAACACC
6846-6871	TCGAGGGATACAACCACGACGACTAC
6956-6930	GGACTGTCATATAGCTACCAGCGAGTC
7134-7156	CAAGCCGAACAAGCTTCCGTTGG
7258-7234	GAGTCGTGGACTCGATAACTACCTC
7818-7839	GTCACCACGGACGAGAAGTAGC
7941-7954	GCGGCGTTCGAAGGATGGCGGCGCGG
7995-8018	CCAACTTCAATCGAGACGACTGCC
8001-7986	GCGGCGTTCGAAAGTTGGAACGCTCAT
8119-8102	GTCAACTGACCGGCCTTC
8249-8225	GTAAATCGTCGGGTCGTCTTCGTTG
10081-10101	GGAGTTCGGTTCTGGTTCCAG
10386-10408	CATAGCGTCCAGACTGTTGAACC
10471-10451	TCGTCAGCGAGGTGAAACAGG
13210-13225	ATAGCGATCGATATGAGTACGCTCGCTG
15568-15589	GTTCGGAGCTGTCACAATCACC
15629-15606	CCATCTTCTGAAGCATGAACACGG
16210-16233	ATCGAGCCACGGAGCTTCAACGAC
16496-16509	GCGGCGTTCGAAGTAAACTCGCTCAT

16530-16507	CATCGACCACCGGAGGTCGGAATG
16542-16521	CATCATTCTCACCATCGACCAC
16580-16567	ATAGCGTTCGAAGCTGACAACCTCGG
16892-16911	GCGTGCTGTTCCTCTCGTTC
17030-17045	GCGGCGTTCGAAGTATAACTACTACTG
17067-17054	GCGGCGTTCGAAAGTTATACACTTAG
17171-17184	GCGGCGTTCGAACCTCACTCTAATAG
17211-17186	GCGGTATCTTTAAGTCCTTAGTTCCCG
17217-17233	GCGGCGTTCGAAAAGTCTTTAGATTCGTG
17226-17211	GCGGCGTTCGAACTAAAGACTTAGAATC
17291-17278	GCGGCGTTCGAATCGTCCTCTTTCAT
17647-17667	TTCTACGAGATGGTGGAGCTG
18366-18379	GCGGCGTTCGAACTAAGACTCAAATC
18371-18385	GCGGCGTTCGAACTCAAATCGGTATC
18421-18402	GCGGCGTTCGAACTCCTCACTCTATTAGACGG
18511-18498	ATAGCGTTCGAACCCAAAAACCACCG
18511-18528	GGACGTAGCTCAGCTCGG
18531-18551	AGAGCGTTCGGCTTCTAACCG
18550-18532	GGTTAGAAGCCGAACGCTC
19584-19601	AAGCGACAGGCTGAGCTG
19598-19580	CTCAGCCTGTCGCTTGAAG
19626-19640	GCGGCGTTCGAATGAAGAACGAGTAG
19713-19700	GCGGCGTTCGAATTGACCCTACCCAC
20101-20118	CTTCGACCTGCTCGATGC
20292-20274	CCGGTTGGTTCATCGAAGG
20503-20481	CAGCGAGAGAAGTGAGAGAGTCC
20649-20662	GCGGCGTTCGAAGCCTTCTAAGAATC
20654-20668	GCGGCGTTCGAACTAAGAATCAAATAG
20707-20688	GCGGCGTTCGAACCTCACTCTATTAGACGTGG
20738-20725	GCGGCGTTCGAAGGCAAGAGAAAGAG
20748-20765	CAGTTGGTAGAGCGCCTG
20751-20769	GCGGCGTTCGAATTGGTAGAGCGCCTGACTG
20783-20761	GACTACCTGATTAACAGTCAGGC
20827-20805	GCGGCGTTCGAAATTAAGGGAAGATACCCGACGCC
20828-20850	CTGGACGTGTTCAAGGAGAACAG
20885-20865	GAATCGAACCGCCCTACACTC
20930-20911	GCGGCGTTCGAAAGCTACGGCATAGGTATTTG
21008-20990	GCTGTTGTAGTCGGTGTCG
21080-21103	CGCCTTCATCTCGTTCAAGATTGG
21225-21206	CCGTATCATCCGACTCGTCG
22025-22042	GAGGTACAGGTCGGTTCG
22088-22110	ACGTTCCTGACCGCTGAGATTCC
22185-22165	GATTCCCACGCAAGTTCTTCC

22237-22216	GTCCGACTTTCGGACGTGCTTC
22261-22275	GCGGCGTTCGAAGATGAAGTAGTATAG
22330-22356	CCCGTTCTCAACCAGATGGAAGATACC
22335-22321	GCGGCGTTCGAAACGGGTATGTTCAT
22348-22331	CCATCTGGTTGAGAACGG
22455-22431	GTTGAACCACGAGAGGAACACGGTC
23950-23977	AGGATACAAGAGCCGGTGAACAATCGAG
24115-24091	CGTCGAAGTCGATGAAGATAGTGTC
25089-25103	GCGGCGATCGATATGAGTTACCAACAG
26281-26295	ATAGCGTTCGAAGATAGCTCCGACTC
26359-26374	GCGGCGATCGATATGGAAAACAAAGACG
26372-26359	GCGGCGTTCGAATCTTTGTTTTCCAT
26784-26799	GCGGCGATCGATATGGAAGTTACAGAAC
27153-27169	GCGGCGATCGATATGATGAGTTTTGGAAC
27539-27525	CGGGATCCTTCTATTCACTTCGTCC
30481-30502	GAACAGCCGAAGTACGCTGTTG
30593-30571	GGAACCACGATAACAACGTCAGC
31421-31441	TCGGAGAGGATAGCAACCGAG
31542-31521	GAGAATATCCGCTGAGATTGCG
34644-34662	ACGAGAGGATTGCCGCATC
36920-36933	ATAGCGTTCGAAGGAGGTCTACGAGG
37071-37058	GCGGCGTTCGAATTCGCTGTCTTCAT
37149-37168	GGACGTTGGTGGTTTCGCTG
37248-37272	GACTGTTGGAGCCACCTCAGTAGTC
37273-37288	GCGGCGTTCGAACCAAAGAGTTAAATC
37330-37315	GCGGCGTTCGAACGTCGTATAGTACCAG
37361-37377	GCGGCGTTCGAAAACGCGGGCGAAATATC
37444-37420	GCGGCGTTCGAAGTGGTAAAACTCAAAGTAGG
37578-37558	GGGTCTTCCACTTCTTCTTCTGG
38589-38607	CCCGATGAACATGGAAGAC
38759-38736	GCAGAGTACGTTCCGTTGTCGTAC
39063-39045	CCAGTCTCCTCGATGAATC
39093-39112	TCACAATCGAGGCCGACTCC
39381-39403	GTCGGAAGAAGGAGTATCGCCTG
39471-39490	GTGTCTGAGTCTGAGTGGAG
40842-40860	GGTCTCGTCCTTACCAAGG
40931-40945	GCGGCGTTCGAACTCTGTACCAGCTAC
40953-40967	GCGGCGTTCGAACCTATTTATAAATAA
41001-40983	GCGGCGTTCGAAGTCCACAGACGGCATACGG
41003-40985	GTGTCCACAGACGGCATAC
41034-41021	GCGGCGTTCGAATCATTTCCCCACAT
41105-41125	CACCTCGCCTTCCTCGAACAC
41146-41117	GTCCTTGATTTGTTCCATCTCGTGTTCGAG

41200-41177	CATGCCGAAGATTTCTGCGGCGAG
41234-41253	CAACACAACGGCGACGTTTC
41327-41341	GCGGCGTTCGAAGTCCCATATACAACC
41385-41372	GCGGCGTTCGAAGACGTATCTACCAT
41426-41409	CGAGTCGGGATGCTATCC
47142-47160	CACCTGATTACACGTCTCG
47671-47686	GCGGCGTTCGAAGTGACTACGGACATTG
47681-47662	TCCGTAGTCACAACTGAAAC
47866-47881	GCGGCGTTCGAACTCCTCACTCTAATAG
47921-47907	GCGGCGTTCGAACTAAGAACAAAGTCG
47938-47923	GCGGCGTTCGAACAGACGACTGACAC
47943-47964	GGAATCGGTCGAGTATCTGCTG
48049-48063	GCGGCGTTCGAACATATCAGTTTCAAG
48064-48080	GCGGCGTTCGAATTAAAGACTCAAATAGG
48118-48105	GCGGCGTTCGAACTTCACTCTAACAG
48132-48150	GTGTAACCAACGCCGTTAG
48133-48119	GCGGCGTTCGAACTCGAAAAATCCAT
48167-48145	CCTTGTTAGAGAGGTCACTAACG
48457-48437	CGCATCGAAGTAAACTTCACC
49107-49089	CGCGCATCTCCGTGCTAAG
49920-49933	GCGGCGTTCGAATTTACTCGTGCCAT
49984-50003	GTTTCCGGGTCTGACGTTTC
50002-49989	GCGGCGTTCGAAAAACGTCAGACCCG
50046-50022	GGTGATATTCCGGCATCTTACGAGG
50259-50239	GGGAATGGCAACAGCAACTCC
52401-52422	GGGATTAGCTCCCTGAACGTAC
52540-52520	TCGAGGATGTTCAGCACCTCG
53290-53309	CTAACCAGTTGCTCTACCTC
54785-54809	GAATAACGACCGCCTGCTCGAACTC
54836-54817	GACCTTGCTTCAGTAAGTGG
54917-54946	CGAAACTACTATTAATGGTAAAGGGTTTATATATT
54946-54917	CGAATATATAAACCCTTTACCATTAATAGTAGTTT
55081-55055	GAATCCGTCTATCACGGTGAATGGTAG
69390-69403	GCGGCGTTCGAAGGTTAACCATAAAG
69431-69418	GCGGCGTTCGAACTAAAGACTTAATC
70170-70150	CTATCTCGCGGAAGCGTGAGC
70230-70211	AGCTGACCGAGGAGCTTTCC
74411-74430	CGCATCGCCGTAGAAGTAGG
74451-74470	GCGGCGTTCGAATACGGTCGAAAGAGCGTCAG
74640-74658	GGAGTCGAACCGGCGTAAG
74660-74641	GCGGCGTTCGAACTCTTACGCCGGTTCGACTC
74699-74673	GAGACAGTAGTGTAGTGGTATCATCGG
74730-74710	GCGGCGTTCGAATACGTCAGTTTGGCCGATATG

74881-74899	AGAGACTTCAACCGGCTCG
75638-75660	TTTCATCGTCGGGGTCGAAGCTC
75650-75627	CCCGACGATGAAATATTCGATGAC
75722-75704	GAACAGCTCGCCGCAGTTG
76444-76467	GGTGTTAGAAAGAGTTGAACTCGG
76602-76630	CGAAACTCCTATTAATGGTAAAGGGTTTATATATT
76630-76602	CGAATATATAAACCCTTTACCATTAATAGGAGTTT
76755-76773	GCGGCGTTCGAAGATAGGCCGATAAGTC
76765-76743	GGCCTATCTTCTTCTTATGGTGC
76816-76803	GCGGCGTTCGAAACCGGGTCTACCAT
77121-77143	TACGAGAGAGGTCAGATGAGGAC
77241-77220	GGTCTGGTTCATCTGGTGTGTG
77380-77356	GCACACGCGCTTTAGTAAGTCCACC

[a]primers are designated by their positions on the HF2 genome (AF222060.2).

Table S3. Oligonucleotide primer used for primer extension on transcripts generated by reporter vector pRV2

Primer	Sequence (5'-3')	Target
Bgal pEXT	GCCATCTGACTGATATCGGTCTCC	214-191[a]

[a]numbering according to pRV2 (accession MZ936313)

Table S4. HF2 infected cell transcripts detected by RT-PCR

Transcript Name	3' end primer	most 5' primer	Locus_tag range (HrrHF2_)[a]	Direction[a]	Estimated length of transcript (bp)[b]
F1	2937-2924	1855-1868	005	+	1,095
F2	15629-15606	3073-3093	010-200	+	12,743
F44	20503-20481	17171-17184	220-255	+	3,413
F52	39063-39045	20751-20769	260-435	+	18,701
F92	77241-77220	41327-41341	460-660	+	35,961
R131	41327-41341	76630-76602	655-460	-	35,320
R90	39471-39490	41003-40985	450-440	-	1,564
R52	1855-1868	20707-20688	260-005	-	18,865

[a]locus tag and direction are according to accession AF222060.2. [b]estimated from the 3' end of the gene in which the cDNA synthesis primer is situated to the 5' end of the gene in which the last productive PCR was detected.

Table S4. Transcript names are as shown in Figure 3 of the main text. The 3' and 5' primer coordinates and locus tags correspond to the HF2 genome sequence (accession AF222060.2).

Supplementary text S1. *Haloferacalesvirus* isolate Hardygib1.

Hardygib1 was isolated in 1998 from Lake Hardy, Victoria (35° 04' S, 141° 44' E) by plating a water sample on lawns of *Haloferax gibbonsii* Ma2.38[T]. Methods of isolation, plaque purification, preparation of virus stock and its long-term storage, DNA extraction and sequencing were as described in [2,3]. The reads were assembled from the same dataset that was used to determine the sequence of the *Haloferax gibbonsii* provirus Halfgib1 [2]. The 73,193 bp Hardygib1 genome sequence is about 96% complete but is missing sequence at both termini. It is estimated to be missing about 1kb at the left end (relative to halovirus HF2, accession AF222060.2), which includes the left terminal direct repeat. At the right end, it is estimated to be missing about 2.5 kb, which includes the right terminal direct repeat, the four genes corresponding to HrrHF2_645 to HrrHF2-660 as well as the N-terminal half of HrrHF2_640. The sequence has been deposited at Genbank under the accession OK649958. Pairwise comparison of viruses available in Genbank (accessed August 2021) shows it to be most similar to HRTV5 (83% nucleotide identity), and its encoded glycine-rich adhesin (corresponding to HrrHF2_490) corresponds to group 3 of [4].

References

1. Porter, K.; Russ, B.E.; Yang, J.; Dyall-Smith, M.L. The transcription programme of the protein-primed halovirus SH1. *Microbiology* **2008**, *154*, 3599-3608.

2. Dyall-Smith, M.; Pfeiffer, F.; Chiang, P.W.; Tang, S.L. The novel halovirus Hardycor1, and the presence of active (induced) proviruses in four haloarchaea. *Genes (Basel)* **2021**, *12*, 149.
3. Dyall-Smith, M.; Tang, S.L.; Russ, B.; Chiang, P.W.; Pfeiffer, F. Comparative genomics of two new HF1-like haloviruses. *Genes (Basel)* **2020**, *11*, 405.
4. Liu, Y.; Demina, T.A.; Roux, S.; Aiewsakun, P.; Kazlauskas, D.; Simmonds, P.; Prangishvili, D.; Oksanen, H.M.; Krupovic, M. Diversity, taxonomy, and evolution of archaeal viruses of the class *Caudoviricetes*. *PLoS Biol.* **2021**, *19*, e3001442.
